# Müllerian papilloma: two case reports of malignant transformation and literature review

**DOI:** 10.3389/fonc.2025.1573747

**Published:** 2025-07-31

**Authors:** Sirong Tao, Yan Zhang, Wei Wang, Ying He, Lili Jiang

**Affiliations:** ^1^ Department of Pathology, West China Second University Hospital, Sichuan University, Chengdu, China; ^2^ Development and Related Diseases of Women and Children Key Laboratory of Sichuan Province, West China Second University Hospital, Sichuan University, Chengdu, China; ^3^ Department of Pathology, West China Hospital, Sichuan University, Chengdu, China

**Keywords:** Müllerian papilloma, malignant transformation, next-generation sequencing, case report, literature review

## Abstract

**Background:**

Müllerian papilloma is a rare benign genital tract tumor, and its malignant transformation is extremely rare. Due to its complex and diverse pathological morphological manifestations, it is prone to misdiagnosis.

**Methods:**

We reported the malignant transformation of Müllerian papilloma into endometrioid carcinoma in two young girls, along with their pathological results. For the first time, we combined next-generation sequencing (NGS) technology to explore the molecular characteristics.

**Results:**

The two cases of malignant transformation into endometrioid adenocarcinoma exhibited similar pathological morphology and immunohistochemical (IHC) markers. Morphologically, they presented complex and diverse features. The benign areas showed a mild papillary structure, while the malignant areas displayed complex papillary branches, cribriform patterns, and solid structures, accompanied by hemorrhage, necrosis, and interstitial inflammatory cell infiltration. In terms of IHC, CK7 and EMA were either focally positive or diffusely positive; Vimentin, P16, and SALL-4 were negatively expressed; P53 showed wild-type expression; the ki67 proliferation index was 35-45%. Subsequent sequencing revealed a low tumor mutation burden and stable microsatellites. However, three novel fusion genes were identified.

**Conclusion:**

The malignant transformation of Müllerian papilloma is extremely rare, with complex and diverse morphological manifestations. High vigilance is required during diagnosis to avoid confusion with sarcomas. This tumor has a low tumor mutation burden and stable microsatellites, and the exact mechanism of malignant transformation requires further investigation.

## 1 Introduction

Müllerian papilloma is a rare benign genital tract tumor, commonly located in the vagina and cervix, mainly occurring in prepubertal girls. It was previously referred to as “mesonephric papilloma”, or “benign polypoid tumor”. Until 1981, based on the ultrastructure of these lesions, Ulbright et al. believed it originated from the Müllerian duct (1). Subsequent immunohistochemical characteristics also supported the Müllerian duct origin (2), and the disease name “Müllerian papilloma” was used. Clinically, patients often seek medical treatment due to vaginal bleeding, and a thorough examination must be carried out to check for the presence of malignant tumors at this time.

The malignant transformation of Müllerian papilloma is extremely rare. In 2003, Abu et al. first described a case of Müllerian papilloma malignant transformation into clear cell carcinoma, which occurred in an adult after multiple recurrences (3). Now, in our hospital, two cases of the malignant transformation of Müllerian papilloma into endometrioid adenocarcinoma have been discovered for the first time. Due to the rarity of these diseases and their diverse morphological manifestations, misdiagnosis is very likely in practical work. As far as we know, there is currently no research on the gene level of Müllerian papilloma, and the exact pathogenesis of its malignant transformation is unclear. Here, we present the clinicopathological features and molecular information of these two cases. At the same time, we reviewed the literature to improve our understanding of the disease, avoid misdiagnosis, and provide evidence for its clinical diagnosis and treatment.

## 2 Case presentation

### 2.1 Case1

#### 2.1.1 Clinical presentation

**Figure 1:**
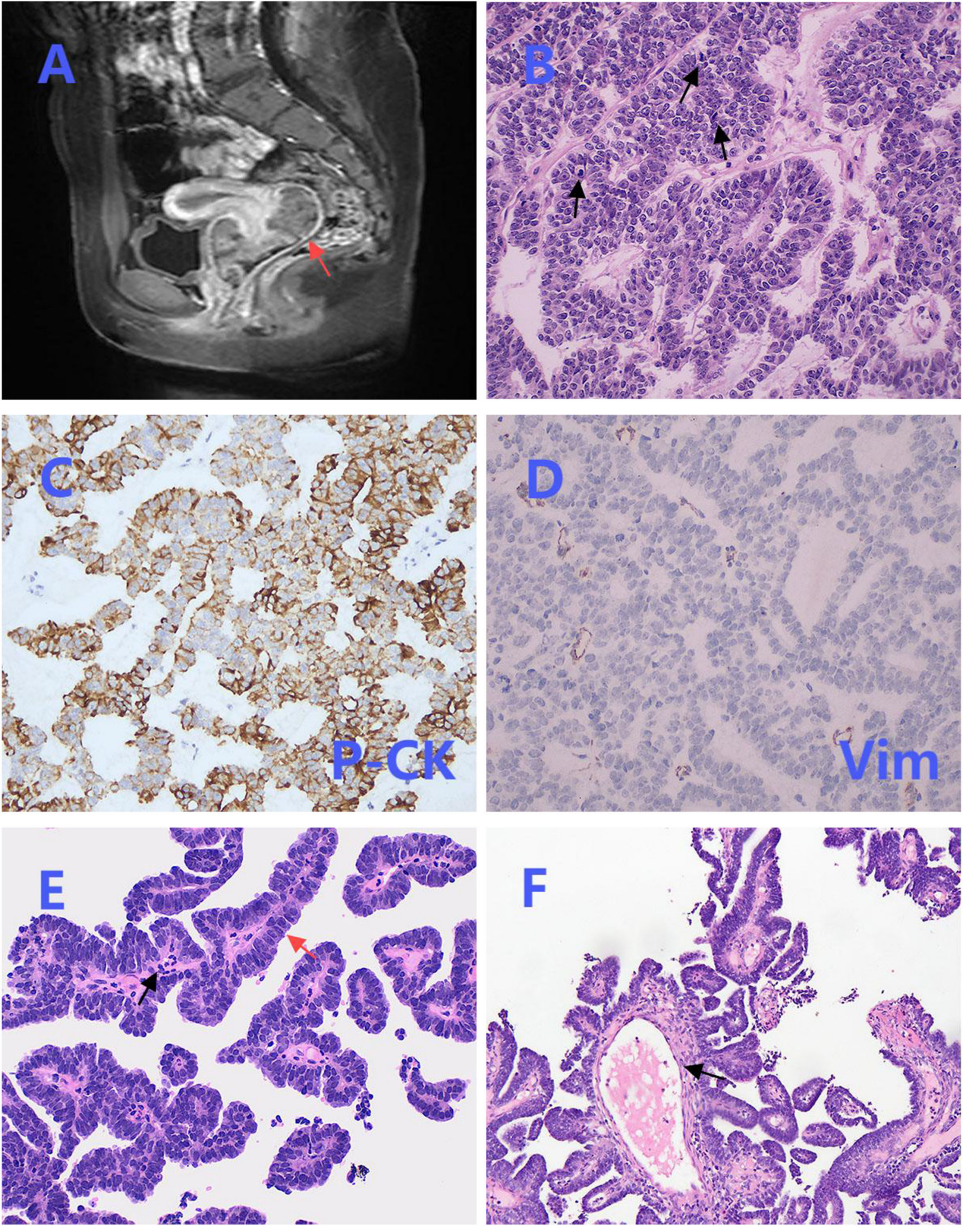
Magnetic resonance imaging (MRI), biopsy and partial postoperative pathological images. **(A)** Pelvic MRI revealed a solid mass at the external os of the cervix, with unclear boundaries (red arrow). **(B)** The tumor cells in the biopsy were arranged in a cord-like and glandular pattern, and mitosis was easily observed (black arrows) (×200). **(C)** Biopsy immunohistochemical staining of P-CK was positive (×200). **(D)** Biopsy immunohistochemical staining of Vimentin was negative (×200). **(E)** The benign area in the postoperative pathology showed a papillary structure, covered by a single layer of columnar epithelium (red arrow), with infiltration of inflammatory cells (black arrow) (×200). **(F)** The fibrous vascular core in the benign area was accompanied by edematous changes (black arrow) (×200).

A 12-year-old girl presented with vaginal bleeding for three months. Her past medical history was unremarkable, and there was no family history of genetic diseases. Physical examination revealed a large mass in the vagina. Pelvic magnetic resonance imaging (MRI) revealed an external cervical solid mass with unclear boundaries, protruding to the upper part of the vagina, with a size of about 7.0cm × 6.2cm × 4.0cm (**Figure 1A**). Routine hematological and biochemical tests showed no abnormalities.

#### 2.1.2 Pathological diagnosis

The patient initially underwent a colposcopic biopsy in the local hospital, which suggested undifferentiated small round cell sarcoma. Subsequently, after pathological consultation in our institution, the initial diagnosis was sarcoma with epithelioid differentiation. Biopsy analysis demonstrated that the tumor cells exhibited an epithelioid morphology, characterized by cord-like and glandular arrangements, a mucinous background, and prominent mitotic figures (**Figure 1B**). Immunohistochemical staining revealed positive expression of P-CK (**Figure 1C**) and negative expression of Vimentin (**Figure 1D**).

Given the malignant nature of the tumor, the patient underwent trans-abdominal total hysterectomy, bilateral salpingo-oophorectomy, and pelvic lymph node dissection on October 18, 2024. The final postoperative pathological diagnosis was the transformation of cervical Müllerian papilloma into endometrioid adenocarcinoma.

**Figure 2:**
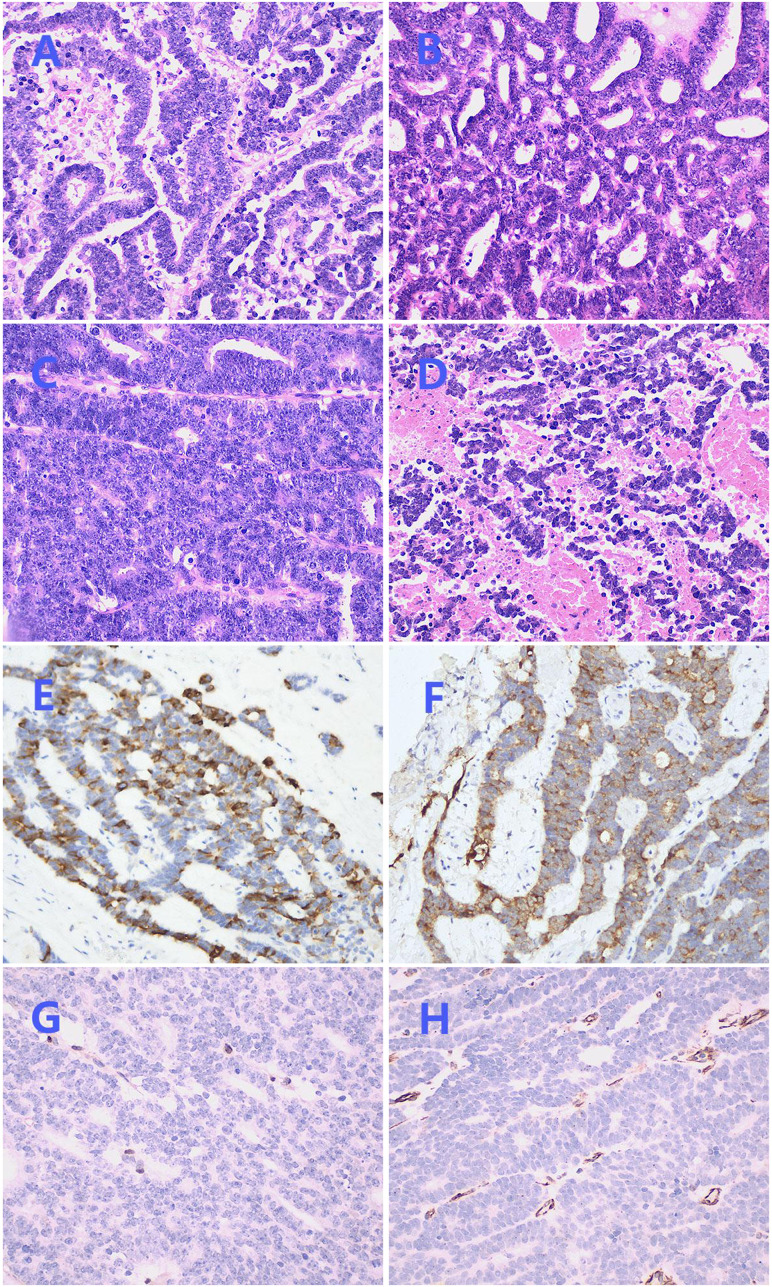
Postoperative pathological images. **(A)** Irregularly shaped glands with stratified columnar epithelium (×200). **(B)** Glands fused into a cribriform structure (×200). **(C)** Solid growth area with enlarged nucleus and obvious nucleolus (×200). **(D)** Focal areas were arranged in a cord-like pattern, accompanied by bleeding and necrosis (×200). **(E)** Immunohistochemical staining of CK7 was focally positive (×200). **(F)** Immunohistochemical staining of EMA was focally positive (×200). **(G)** Immunohistochemical staining of ER was negative (×200). **(H)** Immunohistochemical staining of Vimentin was negative (×200).

Hematoxylin and eosin (HE) staining demonstrated the coexistence of benign and malignant areas within the tumor tissue. The benign area displayed a normal papillary structure, lined by a single layer of columnar epithelium, with no mitotic figures, a visible fibrovascular core, edema-like changes, and chronic inflammatory cell infiltration (**Figures 1E, F**). In contrast, the malignant region exhibited glands arranged in a back-to-back pattern (**Figure 2A**) and fused into a cribriform structure (**Figure 2B**). In some areas, solid growth was observed, with cells showing disorganized arrangement and stratified columnar glandular epithelium characterized by enlarged nuclei and prominent nucleoli (**Figure 2C**). Focal areas of tissue were arranged in a cord-like pattern, with evidence of hemorrhage and necrosis (**Figure 2D**).

Immunohistochemical staining showed CK7 (**Figure 2E**) and EMA (**Figure 2F**) were focally positive, Pax-8 was strongly positive, and PR was weakly positive, while ER (**Figure 2G**), Vimentin (**Figure 2H**), P16, GATA3, CD10, TTF-1, CD56, chromogranin A, synaptophysin, WT-1, Sall-4 were negative, P53 was in wild-type, ki67 index was 45%, no expression loss of mismatch repair proteins was observed. Subsequently, NGS technology was applied to the formalin-fixed paraffin-embedded tissue of the lesion. DNA sequencing showed a low TMB and stable microsatellites. However, in RNA sequencing, we found three new gene rearrangements: MAML1 - KAT6B (EX1:EX17), KAT6B - MAML1 (EX16:EX2), and KTN1 - MAPK1IP1L (EX5:EX4).

#### 2.1.3 Treatment and follow-up

After surgery, the patient received adjuvant radiotherapy. In the most recent follow-up, the patient had no disease recurrence but had post-radiotherapy myelosuppression.

### 2.2 Case2

This case was first reported in our hospital in 2019 (4). Through follow-up and genetic testing, we updated the previous case report. The patient was a 13 - year - old girl who had experienced irregular vaginal bleeding for 2 years. Both digital rectal examination and pelvic MRI revealed a mass within the vagina, and the pathological biopsy suggested rhabdomyosarcoma. After 4 cycles of neoadjuvant chemotherapy, the patient underwent transabdominal total hysterectomy and pelvic lymph node dissection in our hospital on July 28, 2016. Based on the postoperative pathology and immunohistochemistry, the final diagnosis was endometrioid adenocarcinoma resulting from the malignant transformation of Müllerian papilloma in the vagina and cervix. After the operation, the patient received regular adjuvant therapy, including 6 cycles of chemotherapy and 23 sessions of radiotherapy. In the latest follow-up, no disease recurrence or metastasis was found, and there were no obvious chemoradiotherapy reactions. It should be noted that due to the long time elapsed, which affected the quality inspection of the tumor tissue, we did not obtain the genetic test results of Case 2.

## 3 Discussion

Müllerian papilloma is a benign tumor originating from Müllerian epithelium. By reviewing 24 reported cases of benign Müllerian papilloma in the literature (**Table 1**) (1, 2, 5–22), we summarized key characteristics including patient age, symptoms, tumor size, treatment modalities, and outcomes. The majority of cases (23/24, 95.8%) occurred in prepubertal girls with a median age at presentation of 5 years (range, 9 days to 24 years). Lesions were predominantly located in the cervix (12/24, 50.0%) and vagina (11/24, 45.8%), with only one case involving both sites (1/24, 4.2%). Clinical manifestations included vaginal bleeding, discharge, and asymptomatic masses. Diagnosis primarily relied on colposcopic pathological biopsy. Morphologically, classic Müllerian papilloma exhibits a papillary structure covered by single-layer or stratified columnar epithelium or metaplastic squamous epithelium. The stroma was edematous, containing fibrovascular cores and inflammatory cells. Psammoma bodies or bone metaplasia were occasionally observed. The cytoplasm was eosinophilic, with minimal nuclear pleomorphism and mitotic figures. The prognosis was favorable after local resection of the lesion.

**Table 1:** Clinicopathological features of previously reported Müllerian papilloma.

Case no.	References	Age	Symptoms	Comorbidities	Location	Size (cm)	Histology	IHC	Treatment	Outcome
1	Dudič, R. et al. (20)	19m	Vaginal bleeding	Secondary immunodeficiency virus infection	Vagina	2	Classic BMP with acute inflammatory changes	NA	Local excision	Recurrence at 10m,local excision
2	Lucchetti, M. C. et al. (7)	2y6m	Vaginal bleeding	NA	Cervix	NA	Classic BMP	desmin-	Local excision	7y/NED
3	McQuillan, S. K. et al. (14)	5y	Vaginal bleeding	Prolonged activated partial thromboplastin time	Cervix	3	Classic BMP with a mixed inflammatory infiltrate	NA	Local excision	NA
4		7y	Vaginal bleeding	Osteosarcoma	Cervix	NA	Classic BMP	NA	Local excision	25y/NED
5		9y	Vaginal bleeding	Constipation	Cervix	NA	Classic BMP	NA	NA	NA
6		10y	Vaginal bleeding	Insulin-dependent diabetes	Cervix	NA	Classic BMP	NA	Local excision	NA
7	Yalamanchili, V. et al. (10)	2y	Vaginal bleeding	None	Anterior vaginal wall	1.3	Classic BMP	NA	Local excision	NA
8	Kumar, A. et al. (9)	4y	Vaginal bleeding	NA	Cervix	NA	Classic BMP	NA	Local excision	NA
9	Smrkolj, S. et al. (15)	19y	Vaginal bleeding	Proteus syndrome, large bilateral ovarian cystadenomas	Cervix	NA	Classic BMP	NA	radical operation	5y/NED
10	Liu, H. L. et al. (5)	4y7m	Vaginal bleeding	NA	Vagina, Cervix	0.2	Classic BMP	NA	Local excision, electrocautery	NA
11	Tumini, S. et al. (12)	9y	Vaginal bleeding	NA	Posterior vaginal wall	NA	Classic BMP	NA	Local excision	NA
12	Reck-Burneo, C. A. et al. (22)	2y	Vaginal bleeding	None	Anterior vaginal wall	4	Classic BMP	NA	Local excision	3y/NED
13	Hollowell, M. L. et al. (11)	1y3m	Vaginal bleeding	NA	Cervix	2.3	Classic BMP,feathering crowded small hyperchromatic cells,with a high nuclear:cytoplasmic ratio	CEA+, desmin、myogenin-	Local excision	1.5m/NED
14	Mierau, G. W. et al. (8)	4y	Vaginal bleeding	NA	Posterior vaginal wall	4.8	Classic BMP	NA	Local excision	4y/NED
15		9d	Vaginal bleeding	NA	Vaginal	0.1	Classic BMP	NA	Local excision	4y/NED
16	Lane, B. R. et al. (16)	1y6m	Vaginal bleeding	Multiple renal cysts and Wilms tumor	Cervix	1	Classic BMP	NA	Local excision	8m/NED
17	Arbo, E. et al. (21)	2y	Vaginal bleeding	NA	Posterior vaginal wall		Classic BMP	CK1、EMA、CEA +, CK2、Vimentin-	Local excision	NA
18	Jin, M. et al. (6)	2y	Vaginal bleeding	NA	Cervix	2.6	Classic BMP	NA	Local excision	NA
19	Cohen M. et al. (17)	13y	Vaginal bleeding	Post Tylenol use	Vaginal	3	Classic BMP	NA	Local excision	NA
20	McCluggage, W. G. et al. (13)	24y	Painful swelling in the vagina	Pregnant	Posterior vaginal wall	4	Within the underlying stroma,classic BMP with necrotic area	CAM5.2、EMA、CEA、CA125+	Local excision	NA
21	Smith, Y. R. et al. (18)	4y	NA	Chronic abdominal pain and diarrhea.	Cervix	NA	Classic BMP	NA	Biopsy	Recurrence at 1y,local excision
22	Schmedding, A. et al. (2)	2y	Vaginal bleeding	NA	Cervix	NA	Classic BMP	CK、E-cadherin+, desmin-	Local excision	6y/NED
23	Lüttges, J. E. et al. (19)	5y	Vaginal bleeding	Whooping cough	Vaginal	0.8	Classic BMP	CK1、EMA、CEA、AE1+, desmin、Vimentin-	Local excision	Recurrence at 2y
24	Ulbright, T. M. et al. (1)	5y	None	NA	Posterior vaginal wall	5	Within the underlying stroma,classic BMP	NA	NA	1y/NED

NA, not available;IHC, immunohistochemical; “+”, positive; “−”, negative; BMP, benign müllerian papilloma; m, month; y, year; d, days.

However, malignant transformation of Müllerian papilloma is exceedingly rare, with only three documented cases (including the current cases) reported previously (**Table 2**). The earliest reported case involved clear cell carcinoma in an adult with severe cerebral palsy (3). In this study, the two cases exhibit similar clinical and pathological features. Compared to benign Müllerian papilloma, the clinical symptoms are comparable, but tumors with malignant transformation tend to occur in older children and present as larger masses with poorly defined borders relative to surrounding tissues. Microscopically, both benign and malignant areas coexisted: the benign area showed mild papillary structures, while the malignant area displayed various architectures, including branched papillary, cord-like, cribriform, and solid structures. Mitotic figures and nuclear atypia were prominent, accompanied by necrosis, hemorrhage, and deep infiltration. Immunohistochemically, due to the low degree of differentiation of cancer cells, CK and EMA markers are typically only focally positive. Studies have shown that Vimentin and ER expression levels in cervical adenocarcinoma are significantly lower than those in endometrial adenocarcinoma (23, 24). Therefore, the negative expression of Vimentin and ER further supports adenocarcinoma of cervical origin.

**Table 2:** Clinical and molecular information of the malignant transformation.

Case no.	Age	Symptoms	Comorbidities	Location	Size (cm)	Histologic features	IHC	NGS results	Clinical stages	Treatment	Outcome
1	12y	Vaginal bleeding	NA	Cervix	7	Benign area: Papillary. Malignant area: Complex branched, cord - like, cribriform, solid structures	Ck7, EMA, PR+; Vim, ER, SALL-4, P16, P53-; ki67 45%	No gene variation, TMB-L, MSS; three novel fusion genes	IIA2	Radical operation, adjuvant radiotherapy	No recurrence until now, but with myelosuppression
2	13y	Vaginal bleeding	Uterine hypoplasia, Anemia	Vagina, Cervix	7.1	Benign region: Papillary. Malignant region: Complexly branched, cribriform, solid structures.	CK, EMA, ER, PR+; Calretinin, Vim, SALL-4, P16, P53-; ki67 35%	NA	IIA2	Neoadjuvant chemotherapy, radical operation, adjuvant radiotherapy and chemotherapy	No recurrence until now
3	52	Vaginal bleeding	Cerebral palsy	Vaginal	NA	Benign region: Papillary. Malignant region:Typical clear cell carcinoma	NA	NA	NA	Local excision	NA

NA, not available;IHC, immunohistochemical; “+”, positive; “−”, negative; NGS, next-generation sequencing; TMB-L, low tumor mutational burden; MSS, microsatellite stability; y, year.

In clinical practice, diagnosing malignant transformation of Müllerian papilloma is extremely challenging. First, cancers in young girls are rare, leading to cautious consideration of such diagnoses. Second, initial biopsies may not be fully reliable, as areas of small-round cells arranged in cords and mucus background can be mistaken for sarcomas, particularly embryonal rhabdomyosarcoma, a common cervical malignancy in children (25). Immunohistochemical analysis revealed positive expression of P-CK, whereas other markers, including P63, CK7, and Vimentin, demonstrated negative results. Consequently, during the initial biopsy evaluation, considering the patient’s age, morphological characteristics, and immunohistochemical findings, a sarcoma was highly suspected. Postoperative pathological examination of our two cases uncovered diverse morphological features. However, biopsies often represent localized lesions, which may be confounded by necrosis and hemorrhage, thereby complicating accurate diagnosis. Therefore, multi-site biopsies and meticulous microscopic observation are essential to enhance diagnostic accuracy.

Müllerian papilloma malignant transformation into endometrioid carcinoma is extremely rare, and it usually needs to be differentiated from other tumors of the female reproductive system at the time of diagnosis:

Benign Müllerian papilloma: Morphologically, the papillary structure is regular without fusion or significant atypia, and mitotic figures are uncommon. Immunohistochemically, ER and PR are typically positive, while the cell proliferation index Ki-67 remains low (4).

Mesonephric duct adenocarcinoma: A rare subtype of cervical adenocarcinoma that is HPV-independent. Tumor cells often exhibit a cuboidal or low columnar appearance, arranged in small tubular, cord-like, or solid nest patterns. Occasionally, eosinophilic secretions may be observed within the lumen, which differs from Müllerian-derived tumors regarding cellular morphology and arrangement. Immunohistochemical markers such as GATA3, PAX8, and CD10 (apical and luminal staining) can be positive, while TTF-1 and CEA are rarely positive, ER and WT1 are typically negative, and KRAS gene mutations are frequently present (26, 27).

Neuroendocrine carcinoma: Cervical neuroendocrine carcinoma mostly occurs in adult females. Morphologically, tumor cells show nested, trabecular, or rosette arrangements, with fine chromatin (“salt-and-pepper” appearance). In terms of immunophenotype, it characteristically expresses neuroendocrine markers (such as CD56, chromogranin A, synaptophysin), and due to its frequent association with HPV infection, p16 often shows diffuse positivity.

Serous carcinoma: Primary serous adenocarcinoma of the cervix is extremely rare (28). Both Müllerian papilloma malignant transformation into endometrioid carcinoma and serous carcinoma may display complex papillary structures with varying degrees of cellular atypia and mitotic figures. Serous carcinoma is more prevalent in postmenopausal women, characterized by papillae with numerous slender branches. WT-1 is often positive, and abnormal p53 protein expression and p53 gene mutations are common (29, 30).

Malignant Müllerian mixed tumor: This tumor exhibits bidirectional differentiation, containing both malignant epithelial and mesenchymal components. Epithelial components express markers such as CK and EMA, while stromal components express mesenchymal markers like Vimentin and occasionally sarcoma-specific markers (31).

For treating malignant transformation of Müllerian papilloma, no established guidelines exist, so cervical cancer protocols are referenced (32). In the earliest case of clear cell carcinoma, radical surgery and adjuvant treatments were not pursued due to the patient’s overall condition. In our two cases, both were clinically staged as II A, with favorable prognoses following radical surgery and adjuvant treatments. Case 1 did not receive neoadjuvant therapy before surgery and had bilateral adnexectomy, while Case 2, who had combined anemia, received neoadjuvant chemotherapy before surgery and preserved both adnexa. Through follow-up, no signs of recurrence or metastasis were observed in these two cases. Given the limited number of cases, further research on large cohorts is needed to determine optimal treatment strategies.

In fact, approximately 25% of prepubertal vaginal bleeding events remain undiagnosed (33). Therefore, those cases without a clear diagnosis and asymptomatic cases cannot be actively detected. The mechanism underlying the malignant transformation of Müllerian papilloma remains unclear. It is unknown whether this transformation occurs directly from benign tumors or when the critical point of transition from benign to malignant occurs. If it can be detected early and actively treated before the patient’s malignant transformation, the impact of the lesion on the patient’s reproductive system may be reduced.

In this study, we report the genetic test results of Müllerian papilloma with malignant transformation using NGS technology for the first time. DNA sequencing revealed TMB-L and MSS. RNA sequencing identified three novel fusion genes: MAML1-KAT6B (EX1:EX17), KAT6B-MAML1 (EX16:EX2), and KTN1-MAPK1IP1L (EX5:EX4). These gene fusions have been observed with other partner genes. Studies have found that MAML1/2 promotes the nuclear localization of YAP/TAZ and tumorigenesis (34). In 2023, Warmke LM et al. reported an NR1D1-MAML1-fused epithelioid and spindle cell sarcoma, which was similar to pseudomyogenic hemangioendothelioma (PHE) in core biopsies (35). Zafir et al. proposed that MAML1 is a co-regulator that changes the adhesion ability of endometrial epithelial cells (36). Based on the above studies, we believe that MAML1 may be related to the formation of endometrioid adenocarcinoma in this case. The KAT6B gene encodes histone acetyltransferase, which regulates gene expression by modifying lysine residues on histones, thereby affecting the structure of chromatin (37). Abnormal function of these genes is associated with the occurrence and development of cancer. Here, we speculate that the formation of the newly formed MAML1-KAT6B fusion gene may be related to the malignant transformation of Müllerian papilloma. Therefore, in the future, a large number of similar cases need to be collected for in-depth research, to further explore the clinical significance of these fusion genes, and to analyze in detail the mechanism of Müllerian papilloma and its malignant transformation, so as to deepen the understanding of the disease and promote its early detection and treatment.

In general, the malignant transformation of Müllerian papilloma is extremely rare, presenting complex and diverse morphologies under the microscope. High vigilance is required during diagnosis, and attention must be paid to the limitations of local biopsy diagnosis to avoid confusion with sarcomas. This tumor has a low tumor mutation burden and stable microsatellites, and the exact mechanism of malignant transformation still needs further study.

## Data Availability

The original contributions presented in the study are included in the article/supplementary material. Further inquiries can be directed to the corresponding authors.
